# Risk of Oral Squamous Cell Carcinoma in One Hundred Patients with Oral Lichen Planus: A Follow-Up Study of Umberto I University Hospital of Rome

**DOI:** 10.3390/cancers15113004

**Published:** 2023-05-31

**Authors:** Gianluca Tenore, Ahmed Mohsen, Federica Rocchetti, Giulia Rossi, Andrea Cassoni, Andrea Battisti, Marco Della Monaca, Cira Rosaria Tiziana Di Gioia, Francesca De Felice, Andrea Botticelli, Valentino Valentini, Carlo Della Rocca, Marco De Vincentiis, Antonella Polimeni, Umberto Romeo

**Affiliations:** 1 Head and Neck Tumor Board, Umberto I University Hospital, Sapienza University of Rome, 00161 Rome, Italy; gianluca.tenore@uniroma1.it (G.T.); ahmed.mohsen@uniroma1.it (A.M.); rossi.1939733@studenti.uniroma1.it (G.R.); andrea.cassoni@uniroma1.it (A.C.); andrea.battisti@uniroma1.it (A.B.); marco.dellamonaca@uniroma1.it (M.D.M.); cira.digioia@uniroma1.it (C.R.T.D.G.); francesca.defelice@uniroma1.it (F.D.F.); andrea.botticelli@uniroma1.it (A.B.); valentino.valentini@uniroma1.it (V.V.); carlo.dellarocca@uniroma1.it (C.D.R.); marco.devincentiis@uniroma1.it (M.D.V.); antonella.polimeni@uniroma1.it (A.P.); umberto.romeo@uniroma1.it (U.R.); 2 Department of Oral Sciences and Maxillofacial Surgery, Sapienza University of Rome, 00161 Rome, Italy; 3 Department of Radiological Sciences, Oncology and Anatomical Pathology, Sapienza University of Rome, 00161 Rome, Italy; 4 Department of Medico-Surgical Sciences and Biotechnologies, Sapienza University of Rome, 04100 Latina, Italy; 5 Department of Sense Organs, Sapienza University of Rome, 00161 Rome, Italy

**Keywords:** alcohol, malignant transformation, oral cancer, oral lichen planus, oral squamous cell carcinoma, smoking, tobacco

## Abstract

**Simple Summary:**

The malignant transformation risk of oral lichen planus (OLP) is still a dilemma in the literature. The first report of oral squamous cell carcinoma with the origin of OLP was in 1910. Initially, the reported risk in the literature was high after this date; then, with the application of restricted criteria for the diagnosis of OLP, the reported risk become too low. Currently, there is a recommendation to conduct further studies in which other variables are considered, such as smoking and drinking habits and the type and localization of the lesion. This is in order to evaluate the exact risk of OLP and, correspondingly, to avoid the clinical underestimation by medical staff of the risk of malignant transformation of OLP.

**Abstract:**

This study aims to retrospectively assess the potential risk of malignant transformation in patients with diagnosed oral lichen planus (OLP) between 2015 and 2022, and to evaluate the influence of different risk factors. The department’s database and medical records from 2015 to 2022 were searched for patients with a confirmed diagnosis of OLP based on both clinical and histological parameters. A total of 100 patients (59 females and 41 males) were found with a mean age of 64.03 years. In the considered period, the percentage of diagnosed OLP patients was 1.6%, while the percentage of diagnosed OLP patients with transformation to oral squamous cell carcinoma (OSCC) was 0.18%. A statistically significant difference was found with age (*p* = 0.038), tobacco status (*p* = 0.022), and radiotherapy (*p* = 0.041). The analysis revealed the presence of significant risk in ex-smokers (>20 pack-years), with an odds ratio (OR) of 10.0000 (95% confidence interval (95% CI) 1.5793–63.3186); in alcohol-drinker patients, with an OR of 4.0519 (95% CI 1.0182–16.1253); in ex-smoker and alcohol-drinker patients, with OR of 17.6250 (95% CI 2.2464–138.2808); and in patients who had undergone radiotherapy, with OR of 6.3000 (95% CI 1.2661–31.3484). The malignant transformation of oral lichen planus was slightly higher than thought, and the results revealed a possible association with age, tobacco and alcohol status, and history of radiotherapy. An elevated risk of malignant transformation was observed in heavy ex-smoker patients, alcohol-drinker patients, and alcohol-drinker patients with a history of smoking (ex-smokers). Persuading the patient to quit tobacco and alcohol consumption and periodic follow-ups are recommended in general, but particularly in the presence of these risk factors.

## 1. Introduction

Oral lichen planus (OLP) is a chronic inflammatory autoimmune disease that affects the oral mucosa, with clinical manifestations ranging from the presence of white lace-like lesions (reticular form) to plaque-like, erosive, or atrophic lesions [1]. The etiology is still unknown, affecting from 0.5 to 2% of the general population with a higher predilection for middle-aged female patients [2,3].

Histologically, OLP is characterized by the presence of basal-cells degeneration and a subepithelial band-like infiltration by T-lymphocytes that result from a T-cell-mediated response against epithelial basal cells [4]. Clinically, OLP can be manifested in six different subtypes that can be categorized into two groups. The first group comprises the non-erosive-atrophic forms, which are usually asymptomatic forms and include the reticular form (the most common form), papular form, and plaque-like form. The second group comprises the atrophic-erosive forms, which are symptomatic forms that include pain, burning, or soreness in a spontaneous way or during chewing or toothbrushing. This group includes bullous, atrophic or erythematous, and erosive or ulcerative forms [5,6].

According to the majority of clinicians and researchers, the diagnosis of OLP is based on both the clinical and histological criteria of the modified WHO criteria for OLP diagnosis [4]. Primarily, these criteria are the clinical presence of relatively symmetrical white lace-like lesions and the histological presence of liquefaction degeneration at the basal epithelial cell layer, a well-defined band of lymphocytic infiltration, and the absence of epithelial dysplasia. Where the above criteria are not fulfilled, the lesions would be considered oral lichenoid lesions (OLL) [4,7].

OLP is considered an orally potential malignant disorder (OPMD) [8]. Many reports and studies have been performed to investigate the potential risk of malignant transformation (MT) of OLP since the first report of possible transformation of OLP to oral squamous cell carcinoma (OSCC) by Hallopeau et al. in 1910 [3,9]. However, the malignant rate is still controversial. In the beginning, several studies reported MT rates between 0 to 12.5% [3]. Then, some authors noted the loose following of the diagnostic criteria of OLP and suggested that this rate was too high [3,10]. After that, several studies followed these suggested strict criteria by excluding the OLLs, erythroplakia, proliferative verrucous leukoplakia (PVL), and the so-called lichenoid dysplasia [3,4]. Correspondingly, the reported MT rates decreased considerably, with a range from 0.07% to 5.8%, and some studies showed no MT risk [5].

However, the dilemma of the actual MT rate of OLP remains in the literature, because some authors believe that the exclusion of epithelial dysplastic lesions and considering that as a gold standard for the diagnosis of OLP would definitely decrease the MT rate, and these may lead to the underestimation of the potential risk of the MT rate of OLP [5,8,11]. Some authors noted that many studies evaluating the MT rate of OLP neglected or did not provide precise information about some important variables that may impact the MT rate; these include oral risk factors and habits of OSCC such as tobacco and alcohol consumption, type and site of the lesions, and the role played by therapy [3,8]. In addition, geographical and circumstantial variables have been reported as possible factors that may influence the prevalence of OLP and may consequently influence the MT rate [11]. Moreover, the type of specialist and the center in which the diagnosis and management of OLP have been achieved have been reported as variables that influence the prevalence of OLP and may be also factors that influence the MT rate [11].

This study aims to retrospectively assess the potential risk of MT in patients with diagnosed OLP between 2015 and 2022, and to evaluate the influence of different risk factors, including demographic factors, lesion site and type, tobacco and alcohol consumption, diabetes mellitus (DM), hepatitis, thyroid diseases, hypertension, solid or hematological tumor history, and other pathologies.

## 2. Materials and Methods

A single-center retrospective study was carried out on patients referred to the MoMax (Oral Medicine and Maxillofacial Surgeon) project of the Department of Oral Sciences and Maxillofacial Surgery, Sapienza University of Rome. The MoMax project is a created task force of specialists in different medical disciplines of “oral medicine, prosthesis, maxillofacial surgery, oncology, radiotherapy, and histo-pathology”, who collaborate to provide patients with multidisciplinary team care.

All the patients in the study signed an informed consent for participation in the research. All the study procedures were performed in accordance with the ethical standards of the institutional and/or national research committee and with the 1964 Helsinki declaration and its later amendments or comparable ethical standards.

### 2.1. Diagnosis and Management Procedures for OLP Patients

At the first visit, patients with clinical features of OLP were subjected to single or multiple incisional biopsies for the histological confirmation of OLP. After the clinical and histological confirmation of the diagnosis of OLP, the patients were subjected to periodic follow-ups. Photographs were taken at each follow-up visit using the same equipment (Nikon D7500 and AF-S MICRO NIKKOR 105 mm 1:2.8 G, Nikon Corporation, Tokyo, Japan) for the observation of a possible transformation or change of the disease. Based on the characteristics of the disease, the timing of the follow-ups was scheduled with a minimum of two follow-ups per year in the case of stable cases. In the case of the presence of erosive and/or atrophic lesions (acute phase), topical corticosteroid gels and prophylactic anti-fungal mouthwash were prescribed, and follow-ups were increased until the disease was stabilized and the chronic phase was attained. Clinical examination of the oral cavity, evaluation of the status of the documented lesions, and updating the patient’s medical record were performed at each follow-up. Single or multiple incisional biopsies were planned where modification of the clinical features was observed.

### 2.2. Data Collection

The department’s database and medical records were searched from 2015 to 2022. The inclusion criteria were patients with (1) a confirmed diagnosis of OLP based on both clinical and histological parameters, according to the proposed diagnostic criteria of the American Academy of Oral and Maxillofacial Pathology (AAOMP); (2) a follow-up of more than 3 months; and (3) age ≥ 18 years [4,12]. The exclusion criteria were (1) patients with other OPMDs, (2) patients with any clinical and histopathological features of OLLs, (3) patients who were not subjected to histopathological examination, and/or (4) with age < 18 years.

The following data were collected from the department’s database and medical records: age, gender, tobacco status and consumption amount, alcohol status and consumption amount, type and site of lesions, and medical history, including DM, hepatitis, thyroid diseases, hypertension, solid or hematological tumor history, chemotherapy history, radiotherapy history, and other pathologies, if present.

Three categories for the tobacco status of patients were established: non-smokers, ex-smokers, and smokers. The data on tobacco consumption amount was collected from the medical records and was converted into pack-years. Then, the smoker and ex-smoker patients were categorized into two established groups: ≤20 pack-years and >20 pack-years.

Two categories for the alcohol status of patients were established: non-alcohol-drinker patients and alcohol-drinker patients. The type of drink was noted. Information on the amount of alcohol consumption was collected from the medical records and converted into drinks-per-week. The patients were then categorized into two established groups: ≤7 drinks/week and >7 drinks/week. Alcohol abuse issues were documented where present.

The clinical features of OLP at the first visit were retrieved from the patient’s medical records, where the patients were categorized into three categories: patients with only white lesions, patients with any atrophic lesions without erosive lesions, and patients with any erosive lesions with or without other clinical features considered (white and atrophic lesions). The sites of the lesions were also registered, where the oral cavity was defined as follows: buccal mucosa, tongue, the floor of the mouth, palate, and gingiva.

The total follow-up period, the number of follow-ups per year, and the total number of performed biopsies during the follow-up period were retrieved from the patient’s medical records. In addition, the total number of prescribed topical corticosteroids was also documented. 

The cases of MT to OSCC were documented, including the degree of differentiation and site of involvement. The cases of OSCC were considered to be developed from OLP based on the proposed criteria of Krutchkoff et al., Gandolfo et al., and Idrees et al. [10,13,14]. The MT was registered when an OLP patient showed OSCC development at a previously verified lesion and after a follow-up duration of at least 3 months before the development of OSCC. The site of OSCC development in OLP patients was documented using the 10th edition of the International Classification of Diseases (ICD-10), version 2010 [15]. The description of the oral cavity was as follows: tongue (C02.3), superior alveolar ridge mucosa (C03.0), palate (C05.9), and buccal mucosa (C06.0).

### 2.3. Statistical Analysis

The sample size was assessed with a power analysis (G*Power) to define the minimum number of patients necessary to provide a statistical significance of alpha = 0.05 at 80% power. The power analysis results indicated a minimum number of 90 patients with three degrees of freedom and 65 patients with one degree of freedom.

An Excel sheet was created for the retrieved data using Office 365 (Microsoft Corporation, Redmond, WA, USA). The SPSS statistical processing software (Statistical Package for Social Science, Armonk, NY, USA) for Windows, release 25.0, was used for the statistical analysis.

All the considered variables in the study were subjected to descriptive analysis. The variables were as follows: age, gender, tobacco status and consumption amount, alcohol status and consumption amount, medical history (DM, hepatitis, thyroid diseases, hypertension, solid or hematological tumor history, chemotherapy, and radiotherapy), total follow-up period, number of follow-ups per year, number of performed biopsies, number of times prescribed topical corticosteroids, and the grade, site, and timing of OSCC development.

The associations between the presence of MT and the other categorical variables considered in the study (including gender, age range, tobacco status, tobacco consumption amount, drinking status, alcohol consumption amount, DM, hepatitis, thyroid diseases, hypertension, solid or hematological tumor history, chemotherapy, and radiotherapy, OLP clinical type and site, topical prescription of corticosteroids) were evaluated by the chi-square test or Fisher’s exact test in the case of 2 × 2 tables. For all associations, the odds ratio was calculated with a 95% confidence interval (95% CI). Furthermore, *t*-tests for independent samples were used to check for any significant differences in the mean scores of those who showed or did not show MT to OSCC in relation to the number of follow-ups per year and the number of times they were prescribed topical corticosteroids. A statistically significant difference was considered when the *p*-value < 0.05.

## 3. Results

### 3.1. General Preview of the Included OLP Patients

**Figure 1:**
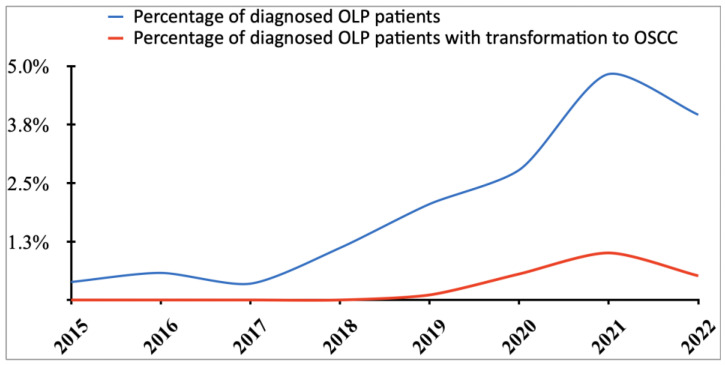
Percentage of diagnosed oral lichen planus (OLP) patients and the percentage of patients with transformation to oral squamous cell carcinoma (OSCC) over the considered 7 years of the study.

Out of 6118 patients, one hundred patients (mean age of 64.03 years) with confirmed clinical and histological diagnoses of OLP were found who fulfilled the inclusion criteria of the study in the considered period (from 2015 to 2022). The percentage of diagnosed OLP patients was 1.6%, while the percentage of diagnosed OLP patients with transformation to OSCC was 0.18%. An obvious increase in both diagnosed OLP patients and OLP patients with transformation to OSCC was observed in the 2021 year, where the percentage was 4.83% and 1.01%, respectively (Figure 1). The patients were distributed into 59 females with a mean age of 65.07 years and 41 males with an average age of 62.71 years. The majority (52%) of patients were >65 years old. 

Forty-five patients had a smoking habit and were distributed as follows: 24 ex-smokers and 21 smokers. Six smoker patients (30%) stopped their smoking habit completely after the diagnosis of OLP and with the provided tobacco counselling by our department health care. For ex-smoker OLP patients, the average tobacco consumption was 21.18 ± 26.7 pack-years. The average smoking duration was 21 ± 15 years, while the average number of years of stopping smoking was 23 ± 14 years. For smoker OLP patients, the average tobacco consumption was 25.98 ± 24.82 pack-years. The average smoking duration was 31 ± 15 years.

Patients with drinking habits comprised 15 patients. The majority of them (11 patients, 73%) reported alcohol consumption of one drink per week or less (≤7 drinks/week). The average alcohol consumption was 8 ± 5 drinks/week. Wine was the reported alcoholic beverage in all the alcohol-drinker patients. 

For the medical history analysis, a total of 42 patients had hypertension, 25 patients had thyroid diseases, 21 patients had cardiological and hematological disorders, 21 patients had a solid or hematological tumor, 12 patients had DM, 8 patients had been subjected to radiotherapy, 4 patients were seropositive for hepatitis C virus (HCV), 3 patients had been subjected to chemotherapy, and 2 patients had a cutaneous lichen planus. Table 1 shows the general profile of the included patients.

### 3.2. Clinical Characteristics of the Included OLP Patients

The patients showed multiple clinical types and site involvement. The most common clinical type was the white type in 72 patients, followed by the atrophic type in 22 patients, and the erosive type in 6 patients. The most involved oral site was the buccal mucosa, and the least involved site was the floor of the mouth. The maximum follow-up duration was 104 months, and the average was 34.42 months. The average number of follow-ups per year was four follow-ups. The average number of performed biopsies per year was one biopsy. The topical corticosteroid therapy was prescribed for 75 patients. The average number of times of it was prescribed was two times. Table 2 shows the clinical characteristics of the included OLP patients.

### 3.3. OLP Patients with Transformation to OSCC

A total of 11 patients (4 females and 7 males) showed a transformation of OSCC at sites diagnosed with OLP (Table 3). The mean patients’ age was 74.91 years. For the smoking habit, 6 of them (54.55%) were ex-smokers, and 2 of them (18.18%) had a tobacco consumption of more than 40 pack-years. The average number of years of smoking in ex-smoker patients was 25 ± 12.7 years, while the average number of years of stopping smoking was 27 ± 13.8 years. Four patients (36.36%) were alcohol drinkers and one of them (9.09%) was drinking more than one drink per week. The most common clinical type was white lesions, which was seen in 7 patients (63.64%). The oral sites of involvement were the tongue (C02.3), in 5 patients (45.45%); the buccal mucosa (C06.0), in 2 patients (18.18%); the palate (C05.9), in one patient (9.09%); the gingiva (C03.0), in one patient (9.09%); and the both buccal mucosa (C06.0) and palate (C05.9), in 2 patients (18.18%). A total of 6 patients (54.5%) showed a well-differentiated degree of differentiation (G1), while 5 patients (45.5%) had a moderately differentiated degree of differentiation (G2).

Evaluating the association between the presence of MT and other variables, a statistically significant difference was found with age (*p* = 0.038), tobacco status (*p* = 0.022), and radiotherapy (*p* = 0.041). Marginal statistical significance was also noticed with alcohol status (*p* = 0.058). To understand the role of each variable in the risk of transformation of OLP to OSCC, the ORs and their 95% CI were calculated, which revealed the presence of significant risk in the following variables: ex-smokers (more than 20 pack-years), with an OR of 10.0000 (95% CI 1.5793–63.3186, *p* = 0.0145); alcohol-drinker patients, with an OR of 4.0519 (95% CI 1.0182–16.1253, *p* = 0.0471); ex-smoker and alcohol-drinker patients, with an OR of 17.6250 (95% CI 2.2464–138.2808, *p* = 0.0063); and patients who had undergone radiotherapy, with OR of 6.3000 (95% CI 1.2661–31.3484, *p* = 0.0246). In addition, a marginal significance was found with patients drinking less than 7 drinks per week, with an OR of 4.1786 (95% CI 0.8995–19.4103, *p* = 0.0680). The statistical analysis revealed the presence of minor risk with smokers; however, this finding should not be considered because there were no smoking patients with OLP transformed to OSCC, which led to this outcome of the statistical analysis.

There was no significant difference found between the presence of MT and the number of follow-ups per year (t = −0.077, *p* = 0.940), topical corticosteroid therapy (*p* = 0.136), and the number of times of topical corticosteroid prescription (t = −0.613, *p* = 0.490). Table 4 shows the calculated ORs for tobacco and alcohol consumption and medical history.

## 4. Discussion

The prevalence of OLP has frequently been reported to be 2% of the general population. In the literature, this prevalence is still not completely confirmed [2,3,11]. Some authors have noted the presence of some factors that may affect the general prevalence, such as geographical factors and the kind of researchers performing the studies [11]. Interestingly, a recent systematic review found a significantly higher prevalence of OLP reported in studies performed by oral medicine and oral pathology specialists compared with the reported prevalence of OLP in studies performed by dentists and dermatologists [11].

Regarding the overall MT, there was a variation in the calculated MT rate of OLP through the years in the literature. Initially, it was too high, but with the addition of some restricted criteria for excluding common similar lesions such as OLLs, the MT reduced significantly. One of the recent studies reported that the overall MT rate was 1.37% [3]. However, another author recently raised great concern regarding the underestimation of the risk of MT of OLP. The author suggested that the MT rate may be higher than reported and that the low reported rates might have been dependent on the methodological quality of studies that did not follow the criteria of diagnosis of OLP rigorously [16]. In particular, these reported rates did not consider many other factors that may increase the MT rate.

The association between DM and OLP is still controversial in the literature [17]. In our study, a total of twelve OLP patients were found to have DM. The analysis did not reveal the presence of any influence of DM on both the OLP and the possible MT to OSCC. A recent systematic review evaluating the association between OLP and DM found that there was a moderate association between them (OR = 1.87, 95% CI = 1.57–2.34), and this association was stronger in studies carried out on European populations compared with studies carried out on Asian populations [18]. In addition, the authors of the study found that the association was stronger with type II DM [17,18]. 

Regarding the MT risk of OLP patients with DM, there were no data showing a possible correlation, and in our study, only one patient showed an MT to OSCC, which was without a statistical significance [5]. Regarding the clinical type, the majority of OLP patients with DM manifested with the white “reticular” type (8 patients (66.7%)), while the atrophic type was observed in only 4 patients (33.3%). This finding was in line with previous studies, where a higher prevalence of the reticular type was reported among OLP patients with DM [17,18].

The association of hepatitis C virus (HCV) with OLP was studied in a systematic review that was carried out on case-control studies examining the prevalence of anti-HCV antibodies in the serum of OLP patients. The study revealed the presence of a statistically significant difference between the OLP patients with HCV seropositivity and controls (OR = 6.07, 95% CI= 2.73–13.48) [19]. Therefore, the necessity for HCV screening in OLP patients has been increasingly recommended [8,13,19]. In our study, only four OLP patients were HCV-positive and the analysis did not show a significant correlation.

The risk of MT to OSCC in OLP with HCV positivity has been also investigated in the literature. In a recent systematic review, 6 studies were analyzed, in which 2160 OLP patients were evaluated, and the analysis of these studies revealed the presence of close to a significant risk of MT to OSCC in OLP patients with HCV positivity (Relative risk (RR) = 4.46, 95% CI = 0.98–20.22, *p* = 0.053) [8]. In our study, only one OLP patient with MT was HCV-positive and the analysis did not reveal the presence of a significant risk (OR = 2.8667, 95% CI = 0.2717–30.2410, *p* = 0.3810).

Many studies have demonstrated smoking and alcohol consumption habits as potential risks for the development of oral cancer [20,21,22]. In our study, the analysis revealed the presence of potential risk of MT to OSCC in ex-smoker patients (>20 pack-years), in alcohol-drinker patients, and particularly in alcohol-drinker patients with a history of smoking (ex-smokers). In the literature, it has been debated as to whether tobacco consumption and drinking habit can be independent causes of the development of OSCC or whether they can be the cause of increasing the risk of transformation of OLP to OSCC [3,5,8]. Some studies considered smoking and drinking habits as confounding factors and excluded OLP patients with these criteria. Correspondingly, the reported MT rates were reduced remarkably. According to the performed analysis in four systematic reviews, smoking and alcohol consumption habits were associated with an increased risk of MT of OLP to OSCC. The authors of all these studies recommended including these factors in future studies as factors that may increase the risk of transformation of OLP to OSCC [3,5,8,13].

Some studies investigated the correlation between thyroid diseases and OLP [23,24,25]. A study by Zhou et al. showed the presence of a higher prevalence of thyroid diseases in OLP patients in the Chinese population (72.4%) that was statistically significant compared with OLL patients and controls [24]. The authors observed that Hashimoto’s thyroiditis in OLP patients had an OR = 3.16 (95% CI = 1.87–5.33) and a thyroid nodule in OLP patients showed an OR = 2.31 (95% CI = 1.30–4.09) [24]. In addition, Garcia-Pola et al. reported in their prospective case-control study a lower prevalence of thyroid diseases in OLP patients (15.35%) and also found a significant association between thyroid disorders and OLP (OR = 3.0673, 95% CI = 1.537–6.117, *p* = 0.001) and a specific association with hypothyroidism [23]. A study was carried out to investigate if there was a correlation between Hashimoto’s thyroiditis and OLP. The study revealed a higher prevalence of Hashimoto’s thyroiditis in female OLP patients compared with the general population [25]. In our study, a total of 25 OLP patients had thyroid diseases. They were distributed as follows: 17 patients with hypothyroidism, 3 patients with Hashimoto’s thyroiditis, 3 patients with a thyroid nodule, and 2 patients with hyperthyroidism. 

Only one patient with hypothyroidism showed MT to OSCC. The presence of thyroid disease as a factor that may increase the risk of MT of OLP to OSCC was rarely considered in the studies. In a study by Tsushima et al., thyroid diseases were considered and a prevalence of 5.5% was found, but all the OLP patients with MT were without thyroid diseases [26].

The most frequently reported clinical types of MT of OLP to OSCC are the erosive and atrophic types [5,8,13]. Regarding the localization of lesions, many studies reported it as a potential risk factor [5,8]. The tongue was the most frequent site of MT. In a recent systematic review, a significant risk of MT was observed with lesions on the tongue compared with other oral sites [8].

One of the recorded patients’ characteristics was radiotherapy. A total of eight OLP patients had a history of radiotherapy. The analysis revealed the presence of a potential risk of MT in OLP patients with a history of radiotherapy (OR = 6.3000 (95% CI = 1.2661–31.3484)). To our knowledge, there is no data regarding the correlation between radiotherapy and OLP or the influence of radiotherapy on the MT risk of OLP. Interestingly, a search of the literature revealed a reported case of a cutaneous lichen planus after breast radiotherapy [27]. Regarding the risk of MT, a hypothesis arising from this finding is that there is a possible increase in the risk of MT in OLP patients with a history of solid or hematological cancers, where the patients with a history of radiotherapy were obviously cancer patients.

The association between psychological disorders, including depression, anxiety, and stress, and OLP has been reported [28]. In a recent systematic review evaluating the degree of association between psychological disorders and OLP, a significantly higher frequency of OLP with stress was revealed compared with the controls (OR = 3.64, 95% CI = 1.48–8.94, *p* = 0.005) [29]. In our study, an obvious increase in the percentage of newly diagnosed OLP patients and in the percentage of MT was observed. The MoMax project was created in 2014. A gradual increase in the general number of referred patients to our project is predicted as a normal growth of the MoMax project activity. However, the trend of increase in the number of patients was obviously different in 2021.

There are many unknown factors that need to be studied that may explain this obvious increase. In a systematic review, it was observed that there is a lack of numerical data investigating the association between the MT risk of OLP and both infective agents (such as candida, and human papilloma virus) and immunosuppressive agents [5]. The increase in patients’ stress after the Coronavirus disease (COVID-19) lockdown period may also explain this, as stress represents a high-risk factor for the development of OLP. The presence of a possible association between LP and COVID-19 infection or vaccination may also be considered, since a recent systematic review reported that LP could be considered a possible rare complication of COVID-19 infection or vaccination [30]. In addition, other authors reported the difficulty of undergoing follow-up appointments as a possible factor in worsening the clinical situation of OLP [31].

Regarding the localization of the lesions and the clinical type, the most reported site of transformation to OSCC of OLP was the tongue. In a systematic review, a statistically significant association was found with tongue localization, with an RR of 1.82 (95% CI 1.21–2.74). A significant risk was also found with the atrophic erosive clinical type (RR = 4.09, 95% CI 2.40–6.98) [8]. In our study, no association between the localization of the lesions, the clinical type, and the MT was found. Surprisingly, the most frequent clinical type of OLP with transformation to OSCC was the white “reticular” type. This finding may highlight the importance of precise periodic follow-ups without taking into consideration the type of lesion. 

There were two observed limitations that should be acknowledged for the proper interpretation of the results. The first was the nature of the study as a single-center retrospective study, which was also from a specialist center of oral medicine and oral pathology. Thus, the results represented our experience and cannot be considered as results of an epidemiological study. The second was the absence of a control group, whose presence might have given more conclusive results.

## 5. Conclusions

Based on the results of our study, the malignant transformation of oral lichen planus is slightly higher than it was thought to be, and the results reveal a possible association with age, tobacco and alcohol status, and history of radiotherapy. An elevated risk of malignant transformation was observed in heavy ex-smoker patients, in alcohol-drinker patients, and particularly in alcohol-drinker patients with a history of smoking (ex-smokers). Persuading the patient to quit tobacco and alcohol consumption and periodic follow-ups are recommended as general measures, particularly in the presence of risk factors, without taking into consideration the type or site of lesions. Further studies, multicenter cohort studies with long follow-up periods, and/or epidemiological studies are needed to assess different possible risk factors for a better understanding of the exact rate of risk of the malignant transformation of oral lichen planus. 

## Figures and Tables

**Table 1:** General profile of the included oral lichen planus (OLP) patients.

Variable		Subgroup	*n* (%)
**Gender**		Female	59 (59)
		Male	41 (41)
**Age**		<51	15 (15)
		51–64	30 (30)
		>64	55 (55)
**Tobacco status**		Non-smokers	55 (55)
		Ex-smokers	24 (24)
		Smokers	21 (21)
**Tobacco consumption amount (pack-years)**	Ex-smokers	≤20 pack-years	18 (18)
		>20 pack-years	6 (6)
	Smokers	≤20 pack-years	11 (11)
		>20 pack-years	10 (10)
**Alcohol status**		Non-alcohol-drinker patients	85 (85)
		Alcohol-drinker patients	15 (15)
**Alcohol consumption status (drinks/week)**		≤7 drinks/week	11 (11)
		>7 drinks/week	4 (4)
**Tobacco/alcohol consumption**		Non-smokers/non-alcohol-drinker patients	51 (51)
		Ex-Smokers /non-alcohol-drinker patients	19 (19)
		Smokers/non-alcohol-drinker patients	15 (15)
		Non-smokers/alcohol-drinker patients	4 (4)
		Ex-Smokers/alcohol-drinker patients	5 (5)
		Smokers/alcohol-drinker patients	6 (6)
**Medical history**		Diabetes mellitus	12 (12)
		Hepatitis	4 (4)
		Solid or hematological tumor	21 (21)
		Hypertension	42 (42)
		Thyroid diseases	25 (25)
		Cutaneous lichen planus	2 (2)
		Cardiological and hematological disorders	21 (21)
		Chemotherapy	3 (3)
		Radiotherapy	8 (8)

**Table 2:** Clinical characteristics and properties of oral lichen planus (OLP) patients.

Variable	Subgroup	Value
**Clinical type:** *n* (%)	White	72 (72)
	Atrophic	22 (22)
	Erosive	6 (6)
**Site of the lesions (at first visit):** *n* (%)	Buccal mucosa	83 (83)
	Lateral border of the tongue	43 (43)
	Ventral surface of the tongue	19 (19)
	Floor of the mouth	17 (17)
	Palate	43 (43)
	Gingiva	59 (59)
**Follow-up duration:** months	Minimum	4
	Maximum	104
	Average	34.42
**Number of follow-ups per year**	Minimum	1
	Maximum	8
	Average	4
**Number of biopsies per year**	Maximum	4
	Average	1
**Topical corticosteroid therapy:** *n* (%)	Yes	75 (75)
	No	25 (25)
**Number of times of topical corticosteroid prescription**	Minimum	0
	Maximum	5
	Average	2

**Table 3:** Characteristics of oral lichen planus (OLP) patients with malignant transformation (MT) to oral squamous cell carcinoma (OSCC).

Variable		Subgroup	Value
**Age:** mean			74.91
**Gender:** *n* (%)		Female	4 (36.36)
		Male	7 (63.64)
**Tobacco status:** *n* (%)		Non-smokers	5 (45.45)
		Ex-smokers	6 (54.55)
		Smokers	0
**Tobacco consumption amount (pack-years):** *n* (%)	Ex-smokers	≤20 pack-years	3 (27.27)
		>20 pack-years	3 (27.27)
	Smokers	≤20 pack-years	0
		>20 pack-years	0
**Alcohol status:** *n* (%)		Non-alcohol-drinker patients	7 (63.64)
		Alcohol-drinker patients	4 (36.36)
**Alcohol consumption status (drinks/week):** *n* (%)		≤7 drinks/week	3 (27.27)
		>7 drinks/week	1 (9.09)
**Tobacco/alcohol consumption**		Non-smokers/non-alcohol-drinker patients	4 (36.36)
		Ex-Smokers/non-alcohol-drinker patients	3 (27.27)
		Smokers/non-alcohol-drinker patients	0
		Non-smokers/alcohol-drinker patients	1 (9.09)
		Ex-Smokers/alcohol-drinker patients	3 (27.27)
		Smokers/alcohol-drinker patients	0
**Medical history:** *n* (%)		Diabetes mellitus	1 (9.09)
		Hepatitis	1 (9.09)
		Solid or hematological tumor	4 (36.36)
		Hypertension	5 (45.45)
		Thyroid diseases	1 (9.09)
		Cutaneous lichen planus	0
		Cardiological and hematological disorders	2 (18.18)
		Chemotherapy	0
		Radiotherapy	3 (27.27)
**Clinical type:** *n* (%)		White	7 (63.64)
		Atrophic	3 (27.27)
		Erosive	1 (9.09)
**Site of lesions:** *n* (%)		Buccal mucosa (C06.0)	2 (18.18)
		Tongue (C02.3)	5 (45.45)
		Palate (C05.9)	1 (9.09)
		Gingiva (C03.0)	1 (9.09)
		Buccal mucosa (C06.0) and palate (C05.9)	2 (18.18)
**Degree of differentiation:** *n* (%)		G1	5 (45.5)
		G2	6 (54.5)
		G3	0 (0)
		G4	0 (0)

**Table 4:** Odd Ratios (ORs) and their 95% Confidence Intervals (CIs) for the associations between the presence of malignant transformation (MT) of oral lichen planus (OLP) to oral squamous cell carcinoma (OSCC) and tobacco status and consumption, alcohol status and consumption, medical history, site of lesions, and clinical type.

**Variable** **Subgroups**			**OR (95% CI)**	***p*-Value**
**Age**				0.038 *
	51–64 vs. <51		1.5763 (0.0606–41.0280)	0.7843
	>64 vs. 51–64		6.4444 (0.7829–53.0483)	0.0832
	>64 vs. <51		7.1538 (0.3956–129.3737)	0.1828
**Gender**				
	Male vs. Female		2.831 (0.771–10.395)	0.119
**Tobacco status**				0.022 *
	Smokers vs. non-smokers		0.2135 (0.0113–4.0342)	0.3031
	Ex-smokers vs. non-smokers		3.3333 (0.9053–12.2726)	0.0702
	Smokers vs. ex-smokers		0.0662 (0.0035–1.2556)	0.0705
**Tobacco consumption amount** (pack-years)				
	Ex-smokers			0.102
		Ex-smokers (≤20 pack-years) vs. non-smokers	2.0000 (0.4273–9.3601)	0.3787
		Ex-smokers (>20 pack-years) vs. non-smokers	10.0000 (1.5793–63.3186)	0.0145 *
	Smokers			1.0000
		Smokers (≤20 pack-years) vs. non-smokers	0.3992 (0.0206–7.7433)	0.5439
		Smokers (>20 pack-years) vs. non-smokers	0.4372 (0.0224–8.5263)	0.5852
**Alcohol status**				0.058
	Alcohol drinkers vs. non-alcohol-drinker patients		4.0519 (1.0182–16.1253)	0.0471 *
**Alcohol consumption amount** (drinks/week)				1.0000
	Alcohol drinkers (≤7 drinks/week) vs. non-alcohol-drinker patients		4.1786 (0.8995–19.4103)	0.0680
	Alcohol drinkers (>7 drinks/week) vs. non-alcohol-drinker patients		3.7143 (0.3398–40.6044)	0.2822
**Tobacco/alcohol consumption**				
	Ex-Smokers /non-alcohol drinkers vs. non-smokers/non-alcohol-drinker patients		2.2031 (0.4444–10.9216)	0.3335
	Smokers/non-alcohol drinkers vs. non-smokers/non-alcohol-drinker patients		0.3405 (0.0173–6.6871)	0.4782
	Non-smokers/alcohol drinkers vs. non-smokers/non-alcohol-drinker patients		3.9167 (0.3271–46.9008)	0.2811
	Ex-Smokers/alcohol drinkers vs. non-smokers/non-alcohol-drinker patients		17.6250 (2.2464–138.2808)	0.0063 *
	Smokers/alcohol drinkers vs. non-smokers/non-alcohol-drinker patients		0.8120 (0.0391–16.8829)	0.8930
**Medical history**				
	**Diabetes mellitus**			1.0000
		With vs. without	0.7091 (0.0826–6.0899)	0.7540
	**Hepatitis**			0.377
		With vs. without	2.8667 (0.2717–30.2410)	0.3810
	**Solid or hematological tumor**			0.236
		With vs. without	2.4202 (0.6354–9.2184)	0.1952
	**Hypertension**			1.000
		With vs. without	1.1712 (0.3324–4.1267)	0.8058
	**Thyroid diseases**			0.283
		With vs. without	0.2708 (0.0329–2.2301)	0.2246
	**Cutaneous lichen planus**			1.000
		With vs. without	1.5217 (0.0687–33.7130)	0.7905
	**Cardiological and hematological disorders**			1.000
		With vs. without	0.8187 (0.1630–4.1119)	0.8081
	**Chemotherapy**			1.000
		With vs. without	1.0745 (0.0521–22.1606)	0.9629
	**Radiotherapy**			0.041 *
		With vs. without	6.3000 (1.2661–31.3484)	0.0246 *
**Site of lesions**				
	**Buccal mucosa**			1.000
		With vs. without	0.9122 (0.1788–4.6538)	0.9120
	**Tongue**			0.345
		With vs. without	0.3629 (0.1019–1.2922)	0.1177
	**Floor of the mouth**			0.392
		With vs. without	2.0089 (0.4739–8.5167)	0.3438
	**Palate**			0.412
		With vs. without	1.6865 (0.4786–5.9425)	0.4160
	**Gingiva**			0.756
		With vs. without	0.8151 (0.2312–2.8738)	0.7505
**OLP clinical type**				0.789
	Erosive vs. white		1.8571 (0.1891–18.2341)	0.5953
	Atrophic vs. white		1.4662 (0.3453–6.2249)	0.6040
	Erosive vs. atrophic		1.2667 (0.1073–14.9501)	0.8511

* *p*-value < 0.05.

## Data Availability

Data can be accessed from corresponding author upon request.
